# Current Approaches for Predicting a Lack of Response to Anti-EGFR Therapy in *KRAS* Wild-Type Patients

**DOI:** 10.1155/2014/591867

**Published:** 2014-06-18

**Authors:** Tze-Kiong Er, Chih-Chieh Chen, Luis Bujanda, Marta Herreros-Villanueva

**Affiliations:** ^1^Division of Molecular Diagnostics, Department of Laboratory Medicine, Kaohsiung Medical University Hospital, Kaohsiung Medical University, 100 Shih-Chuan 1st Road, Kaohsiung 80708, Taiwan; ^2^Translational Research Center, Kaohsiung Medical University Hospital, Kaohsiung Medical University, 100 Shih-Chuan 1st Road, Kaohsiung 80708, Taiwan; ^3^Center for Lipid Biosciences, Kaohsiung Medical University Hospital, 100 Shih-Chuan 1st Road, Kaohsiung 80708, Taiwan; ^4^Biomedical Technology and Device Research Laboratories, Industrial Technology Research Institute, No. 195, Section 4, Chung-Hsing Road, Chutung, Hsinchu 31040, Taiwan; ^5^Department of Gastroenterology, Donostia Hospital, Biodonostia Institute, Center for Biomedical Research in Network for Hepatic and Digestives Diseases (CIBERehd), Centro de Investigación Biomédica en Red de Enfermedades Hepáticas y Digestivas (CIBERehd), University of Basque Country, 20014 San Sebastian, Spain

## Abstract

Targeting epidermal growth factor receptor (EGFR) has been one of the most effective colorectal cancer strategies. Anti-EGFR antibodies function by binding to the extracellular domain of EGFR, preventing its activation, and ultimately providing clinical benefit. *KRAS* mutations in codons 12 and 13 are recognized prognostic and predictive biomarkers that should be analyzed at the clinic prior to the administration of anti-EGFR therapy. However, still an important fraction of *KRAS* wild-type patients do not respond to the treatment. The identification of additional genetic determinants of primary or secondary resistance to EGFR targeted therapy for further improving the selection of patients is urgent. Herein, we review the latest published literature highlighting the most important genes that may predict resistance to anti-EGFR monoclonal antibodies in colorectal cancer patients. According to the available findings, the evaluation of *BRAF*, *NRAS*, *PIK3CA*, and *PTEN* status could be the right strategy to select patients who are likely to respond to anti-EGFR therapies. In the future, the combination of those biomarkers will help establish consensus that can be introduced into clinical practice.

## 1. Introduction

With a global increasing incidence of more than one million cases annually and status as the third most common cancer, colorectal cancer (CRC) is a major health burden [1, 2]. Important progress has been made in the treatment of this disease since the introduction of new therapies that have improved patient survival even after metastasis development. Targeting epidermal growth factor receptor (EGFR) has been intensively pursued as a cancer strategy. In the clinical setting of CRC, the use of monoclonal antibodies to block EGFR has demonstrated important clinical benefit exhibiting antitumor activity as monotherapy or in combination with chemotherapy and/or radiation. In particular, the antibodies cetuximab (IMC-C225, Erbitux) and panitumumab (Vectibix) work by binding to the extracellular domain of EGFR and preventing its activation. Mechanistically, both antibodies prevent EGFR receptor activation and dimerization and ultimately induce receptor internalization and downregulation [3].

## 2. Structure of KRAS, NRAS, BRAF, and PIK3CA Proteins

**Figure 1:**
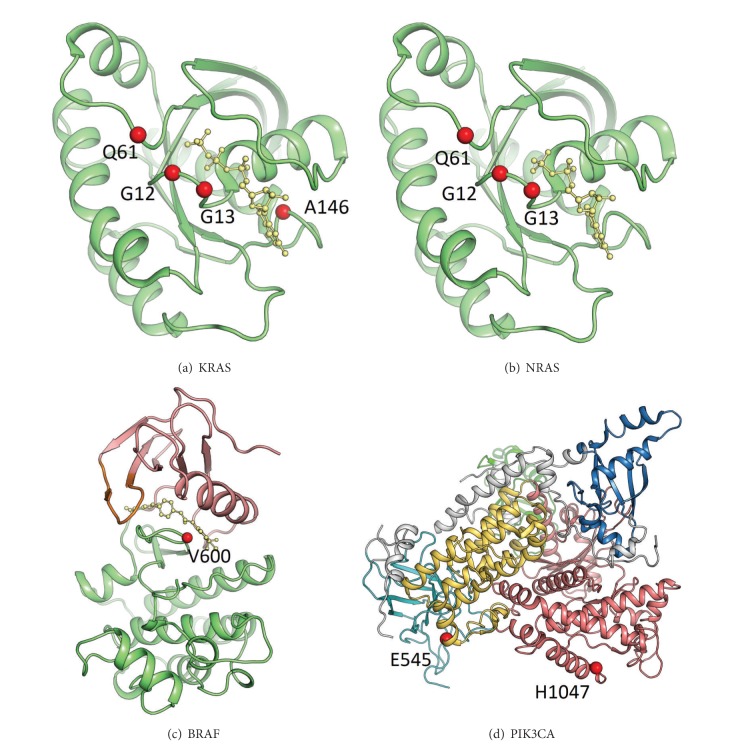
Downstream signaling proteins of EGFR: (a) KRAS, (b) NRAS, (c) BRAF, and (d) PIK3CA. The most frequent activating mutation sites are shown as red spheres.

*KRAS*,* NRAS*, or* BRAF* mutations can all activate the RAS-RAF-MAPK pathway, which is downstream from EGFR. The KRAS and NRAS hotspot mutation sites G12, G13, Q61, and A146 are indicated in Figures 1(a) and 1(b) showing as the red spheres. These mutations activate the oncogenic properties of RAS proteins and it has been reported that they do so by inhibiting GTPase activity. The BRAF hotspot mutation, V600E, located at the A-loop is highlighted in red spheres (Figure 1(c)). This mutation may disrupt an inactive conformation of BRAF kinase. Therefore,* BRAF* V600E increases the kinase activity that provides cancer cells with both proliferation and survival signals and promotes them to become tumors in the model system.* PIK3CA* mutations activate the PI3 K-PTEN-AKT pathway, which is downstream from both the EGFR and the RAS-RAF-MAPK pathways. The PIK3CA mutations E545 and H1047 are located at the helical domain and kinase domain of the protein, respectively (Figure 1(d)). Studies showed that mutant E545 inhibits the activity of the catalytic subunit, because it interacts with L379 and A340 of the p85 nSH2 domain. The mutant H1047 has a direct effect on the conformation of the activation loop, changing its interaction with phosphatidylinositol substrates. Notably, Smith et al. [4] found that exon 9, but not exon 20, mutations in* PIK3CA* were associated with* KRAS* mutations. Exon 9 mutations lie in the helical domain of protein and require interaction with GTP bound RAS. Moreover, exon 20 mutations lie in the kinase domain and require p85 binding but are independent of GTP bound RAS [5].

## 3. Potential Biomarkers for Anti-EGFR Therapy

### 3.1. KRAS

It is well known that* KRAS* mutation is the first described and most important factor contributing to anti-EGFR therapies [6].* KRAS* mutations have been reported to be associated with a lack of response to cetuximab and panitumumab and/or poorer survival in chemorefractory metastatic CRC patients in several independent studies [6–9]. The hypothesis is that* KRAS* mutation activates the RAS/MAPK signaling pathway downstream of EGFR independently of ligand binding to the receptor. Based on confirmed preclinical and clinical data, the European Medicines Agency and the U.S. Food and Drug Administration (FDA) have suggested that only* KRAS* wild-type patients should be candidates to receive cetuximab or panitumumab.

Although 40–60% of CRCs are* KRAS* wild-type [10, 11], the response rate to cetuximab in monotherapy is approximately 10% and does not exceed 23% even when combined with chemotherapy. A very recent hypothesis suggested that* KRAS* mutations may not be detected in initial disease because a small number of cells with* KRAS* mutations exist in the presence of a vast majority of wild-type* KRAS* cells. Diaz et al. found that 38% of patients whose tumors were initially* KRAS* wild-type developed* KRAS* mutations that were detectable in their sera after 5-6 months of treatment [12]. Recently, Custodio and Feliu indicated that, in addition to* KRAS*, there are signaling events/molecules downstream of EGFR that can become unregulated [13]. It is therefore necessary to identify the factors that contribute to anti-EGFR resistance in* KRAS* wild-type patients.

### 3.2. BRAF

The detection of* BRAF* mutations is currently included in some clinical laboratory protocols, although it has not been established as routine clinical practice. BRAF is a protein member of the* RAF* family (RAF1, BRAF, ARAF), also regulated by RAS binding.

*BRAF* encodes a serine-threonine protein kinase that is the most important downstream effector of activated KRAS [14]. Mutated BRAF activates a signaling cascade involving proteins in the mitogen-activated protein kinase system, resulting in cell proliferation [15]. Approximately 15% of CRCs have the* BRAF* mutation, and this is an indicator of poor prognosis regardless of the treatment or administration [16]. Most of the* BRAF* mutations associated with cancer are located in exons 11 and 15, coding for the kinase domain. The hotspot mutation is the T-to-A transversion at nucleotide 1796 that corresponds to the V600E mutation. This mutation is predisposed to the inhibition of apoptosis and also aids in increasing invasiveness [17]. It has also been suggested that* BRAF* mutation is a negative prognostic indicator in CRC [18] and a negative predictor of response to EGFR inhibitors, according to results from CRYSTAL, OPUS, and PICCOLO trials [19–21].* BRAF* mutation was also associated with shorter progression-free survival (PFS) and overall survival (OS) [22, 23].* KRAS* and* BRAF* mutations are mutually exclusive in CRC [24, 25]; therefore, the National Comprehensive Cancer Network (NCCN) suggests considering* BRAF* mutation testing when* KRAS* is wild-type [26]. Different studies demonstrated that* BRAF* mutation confers resistance to both cetuximab and panitumumab [25]. Specifically,* BRAF* is responsible for resistance when patients received anti-EGFR therapy in a second or subsequent round of treatment, as shown in several retrospective studies [10, 25, 27, 28]. In contrast, the predictive value of* BRAF* mutations in first line treatment has not been fully demonstrated [18, 29, 30]. A recent study conducted by Saridaki et al. showed lower PFS and OS in BRAF V600E mutated patients compared with wild-type (4.2 versus 11.1 months and 14.3 versus 35.0 months, resp.), although differences were not significantly significant [31]. Due to the poor prognosis of* BRAF* mutated patients and the lack of response to anti-EGFR therapy, rational therapeutic strategies have been directed toward selective RAF inhibitors. For instance, BRAF inhibitors used for melanoma have also been tested against CRC. However, very little clinical benefit was observed, suggesting that the biological behavior in melanoma and colorectal cancer can be different.

### 3.3. NRAS

KRAS and NRAS are highly homologous and closely related to KRAS [32]. Mutant* NRAS* has been reported to have an antiapoptotic function and promotes CRC in an inflammatory context [33]. Unlike* KRAS* mutations, which are commonly seen in CRC,* NRAS* mutations are found in approximately 3–5% of CRCs and occur most commonly in codon 61 rather than in codon 12 or 13 [13, 34].* NRAS* mutations like* BRAF* mutations are mutually exclusive from* KRAS* mutations [26]. Because* KRAS* and* NRAS* mutations are mutually exclusive,* NRAS* mutation testing should be performed when* KRAS* is wild-type. To date, several studies demonstrated that the presence of* NRAS* mutations is associated with a lack of response to cetuximab therapy [21, 24]. Shen et al. showed that 4.19% (26/621) of tumors harbored an* NRAS* mutation and distant metastatic tumors had a higher* NRAS* mutation rate [34]. The authors recommended that* NRAS* mutation detection should be considered before anti-EGFR therapy, especially in* KRAS* wild-type tumors. Recently, Russo et al. found that* NRAS* mutations were identified almost exclusively in patients with rectal cancer and were more common in older patients [35]. They also showed that* NRAS* mutation was not associated with clinical outcomes. However, di Bartolomeo et al. indicated that* KRAS*/*NRAS* wild-type status was the most important predictor of efficacy in terms of PFS in a TEGAFOX-E (cetuximab, oxaliplatin, and oral uracil/ftorafur-UFT) phase II study [36]. Additionally, Douillard et al. reported that additional* RAS* mutations predicted a lack of response in patients with mCRC who received panitumumab-FOLFOX4 [37]. Most recently, Sclafani et al. showed that a significant proportion of* KRAS*/*BRAF* wild-type patients (17%) had* RAS* mutations beyond* KRAS* exons 2-3 (additional* KRAS* mutations in 10.2%,* NRAS* mutations in 6.8%) in retrospective analysis (EXPERT-C trial) [38]. They also found that the addition of cetuximab was associated with higher response; however, this was not statistically significant. Based on the published literature,* NRAS* mutation testing is required before initiating treatment with EGFR inhibitors. Notably, the Medicines and Healthcare Products Regulatory Agency (MHRA) indicates that the evidence of wild-type RAS status (at exons 2, 3, and 4 of KRAS and NRAS) is required before initiating treatment with panitumumab alone or in combination with other chemotherapy for metastatic colorectal cancer (mCRC). Genetic testing for* RAS* gene mutations (in* KRAS* and* NRAS*) beyond the routine analysis of* KRAS* exon 2 will become the standard for selecting patients for anti-EGFR therapy in near future.

### 3.4. PI3 K/PTEN/AKT

Several preclinical studies note the importance of the PI3 K/PTEN/AKT pathway in determining the sensitivity of CRC cell lines to cetuximab.* PI3KCA* mutations, present in 10–25% of CRCs [28, 39–41], have been reported to be “gain of function” mutations activating the PI3 K/AKT pathway.* PIK3CA* mutations, which are located in exon 9 or exon 20, can coincide with* KRAS* and* BRAF* mutations [42] and exert different oncogenic effects including resistance to anti-EGFR therapies. Although the role of the* PIK3CA* mutational status in the anti-EGFR response is still controversial, several published studies agree on the fact that there is a significant negative correlation between* PIK3CA* mutation in codon 20 and the response to anti-EGFR antibodies [39, 43]. In contrast, Prenen et al. [41] did not find this association, and Karapetis et al. concluded that, in chemotherapy-refractory colorectal cancer, neither* PIK3CA* mutation status nor PTEN expression was prognostic, or predictive of benefit from cetuximab [44]. Additionally, recent data suggest that* PIK3CA* exon 20 mutations are associated with poorer PFS, OS, and objective response to anti-EGFR antibodies, whereas patients with mutations in codon 9 are equally responsive to wild-type subjects [45]. Taking these data together, we can conclude that most published literature supports a role of* PIK3CA* exon 20 in predicting resistance to cetuximab and panitumumab, but further studies need to be performed to display clinical significance.

PTEN is a key tumor suppressor gene involved in PI3 K/AKT signaling. The loss of PTEN, by mutation, allelic loss, or epigenetic events results in the persistent activation of PI3K effectors [46, 47]. It has been shown that the loss of PTEN is present in 19–42% of CRCs and often coincides with* KRAS*,* BRAF*, and* PI3KCA* mutations [48]. Preclinical data have shown that PTEN loss confers resistance to cetuximab-induced apoptosis in CRC cell lines [49]. Jhawer et al. [49] also demonstrated that* PI3KCA* mutation and PTEN expression predict a poor response of colon cancer cells to cetuximab. Some groups suggested that the loss of PTEN protein expression is associated with nonresponsiveness to cetuximab [50]. Retrospective studies provided evidence that the loss of PTEN is associated with poorer response to cetuximab [50, 51]. In contrast, Razis et al. did not find association between PTEN protein expression and clinical outcomes in patients treated with cetuximab [52]. However, because there are contradictory results, principally due to protein expression interpretation, further prospective studies are needed to evaluate PTEN expression for its use in the clinical setting.

Although AKT phosphorylation has been correlated with the response to gefitinib [53], its association with the response to cetuximab has not been addressed. In contrast, some reports have shown that p-AKT can modulate the response to anti-EGFR antibodies [54]. In conclusion, there is no clear evidence useful to the clinical setting to support an improved response to anti-EGFR therapies based on AKT phosphorylation.

### 3.5. HER Family Members

Different preclinical data have suggested that the heterodimers of EGFR with other members of the HER family, such as HER2 and HER3, may affect anti-EGFR therapies. Wheeler et al. [55] analyzed resistance in lung cancer and observed that EGFR, HER2, HER3, and c-MET were highly activated in cetuximab-resistant clones derived from lung cancer cell lines. Their data demonstrated the dysregulation of EGFR internalization/degradation and the subsequent EGFR-dependent activation of HER2 and HER3. Furthermore, it appears that HER3 activity, which depends on EGFR and HER2, represents a critical step for cells to overcome cetuximab effects. Additionally, c-MET was highly phosphorylated in the absence of its ligand HGF. A recent paper has shown that the amplification of the MET protooncogene is associated with* de novo* and acquired resistance in wild-type tumors [56].

Large-scale retrospective analyses have been performed to strengthen the role of HER2 as a resistance biomarker in CRC. The frequency of HER2 amplification is similar to other genes such as BRAF and NRAS [42], and the evaluation of the HER2 gene by FISH may in fact be an additional useful test for the identification of mCRC patients who will benefit from anti-EGFR targeted therapies. Martin et al. [57] concluded that patients with an increased HER2 gene copy number show a worse response to anti-EGFR antibodies. In addition, a phase I clinical trial demonstrated that anti-HER2 therapy combined with cetuximab in refractory CRC was associated with antitumor activity, although the combination was not tolerable due to overlapping toxicities [58]. However, HER2 testing needs to be further investigated for future personalized medicine.

### 3.6. MicroRNAs

MicroRNA (miRNAs) are a class of endogenous, short (17–25 nucleotides), noncoding single-stranded RNAs involved in the posttranscriptional regulation of gene expression [59]. miRNA causes either the degradation or the inhibition of translation by binding imperfectly to the 3′-untranslated region of targeted mRNA [60]. Dysregulated miRNAs are associated with CRC development, progression, and therapeutic response [61]. Ragusa et al. demonstrated that the downregulation of members of the Let-7 family was a predictive marker of cetuximab sensitivity [62]. They also showed that miR-146b-3p and miR-486-5p were less abundant in* KRAS* wild-type compared with* KRAS*-mutated tumors. Similarly, two studies implicated the potential role of Let-7 family members in* KRAS* regulation and anti-EGFR therapy sensitivity in CRC [63, 64]. Additionally, Sebio et al. showed a LCS6 polymorphism in the 3′-UTR of KRAS, which is in a binding site for Let-7, may serve as a predictive marker of anti-EGFR treatment in* KRAS* wild-type and* BRAF* wild-type patients [65]. Meanwhile, Pichler et al. showed that low expression of miR-200a is associated with poor survival [66]. Furthermore, Cappuzzo et al. showed that patients highly expressing the miR-99a/Let-7c/miR-125b cluster showed longer PFS and longer OS than patients expressing low levels of the cluster in the* KRAS* wild-type population [61]. They thus concluded that the miR-99a/Let-7c/miR-125b signature may improve the selection of* KRAS* wild-type patients for anti-EGFR therapy. Recently, Pichler et al. indicated that the miR-181a expression level is associated with poor survival in patients with CRC and that miR-181a expression may predict PFS in EGFR targeted therapy [67]. Based on these findings, miRNAs could be used as predictive biomarkers in selecting patients for anti-*EGFR* antibody therapy in the future.

## 4. Conclusion

**Figure 2:**
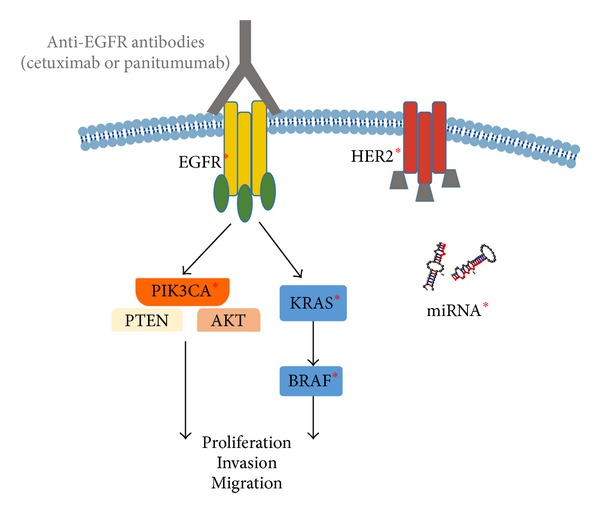
Signaling pathways implicated in the lack of response to anti-EGFR therapies. ∗ indicates some receptors or downstream effectors which are responsible for anti-EGFR resistance when they are mutated or overexpressed.

According to the available data obtained during the last decade, it is clear that the evaluation of not only the* KRAS* mutational status but also* BRAF*,* NRAS*,* PIK3CA*, and* PTEN* alterations could be beneficial to the selection of patients who are likely to respond to anti-EGFR therapies (Figure 2). However, there are no guidelines or recommendations from the European group, United States-based group, or Canadian Expert group recommending the use of* BRAF*,* NRAS*,* PIK3CA*, PTEN, or AKT to select CRC patient for anti-EGFR antibody therapy [68]. Notably, the Evaluation of Genomic Applications in Practice and Prevention (EGAPP) Working Group (EWG) found insufficient evidence to recommend or discourage testing for mutations in BRAF V600E, NRAS, or PIK3CA and/or loss of PTEN or AKT protein. Therefore, the EWG discourages the use of these tests for deciding whether to introduce anti-EGFR therapy with cetuximab or panitumumab until more evidence supports improved clinical outcomes [69]. Moreover, a meta-analysis suggests that mutations in* KRAS* exons 3 and 4,* NRAS*,* BRAF*,* PIK3CA*, and nonfunctional PTEN predict resistance to anti-EGFR therapies [70] and concluded that these biomarkers should be implemented for prediction of clinical benefit from anti-EGFR antibodies in mCRC.

**Figure 3:**
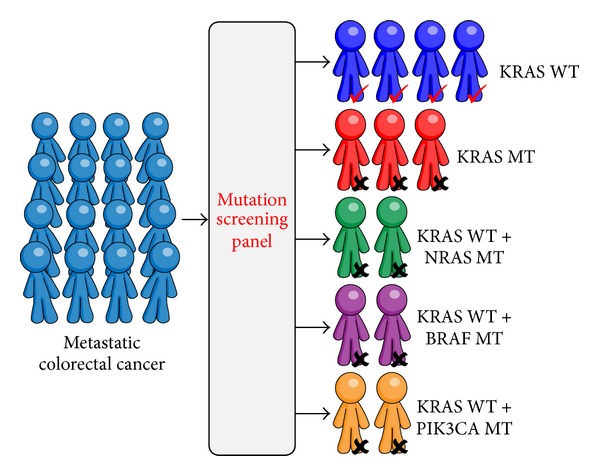
A panel of different genes will be a step forward in the “personalized medicine” of CRC patients for selecting patients and predicting efficacy of anti-EGFR therapy.* KRAS* WT: no mutations were detected in exons 2, 3, and 4.

In the near future, a panel of multiple genes is likely to be analyzed simultaneously and used for selecting patients and predicting the efficacy of anti-EGFR therapy (Figure 3). A panel of these different mutations identifies a subgroup of mCRC patients with distinct biological behavior and response to treatments, including anti-EGFR antibodies. This panel will be a step forward in the “personalized medicine” treatment of CRC patients. In summary, the main molecular markers described in this review may enable an accurate selection of patients who will benefit from anti-EGFR therapy.
